# Dietary and Genetic Aspects of Polycystic Ovary Syndrome (PCOS) in Polish Women—Part II: Association of *CYP19*, *FTO*, *MC4R* and *INSR* Gene Polymorphisms with Clinical Symptoms of PCOS

**DOI:** 10.3390/genes16070840

**Published:** 2025-07-18

**Authors:** Karolina Nowosad, Małgorzata Ostrowska, Paweł Glibowski, Katarzyna Iłowiecka, Wojciech Koch

**Affiliations:** 1 Department of Biotechnology, Microbiology and Human Nutrition, University of Life Sciences in Lublin, 8 Skromna St., 20-704 Lublin, Poland; karolina.nowosad@up.lublin.pl (K.N.); pawel.glibowski@up.lublin.pl (P.G.); 2 Nutrition Clinic, Department of Clinical Dietetics, Medical University of Lublin, 7 Chodzki St., 20-093 Lublin, Poland; katarzyna.ilowiecka@umlub.pl; 3 Department of Food and Nutrition, Medical University of Lublin, Chodźki 4a, 20-093 Lublin, Poland; wojciech.koch@umlub.pl

**Keywords:** PCOS, *INSR*, *MC4R*, *FTO*, *CYP19*, gene polymorphism, acne, Polish women

## Abstract

Background/Objectives: Polycystic ovary syndrome (PCOS) is a multifactorial disorder influenced by both environmental and genetic factors. The aim of this study was to evaluate associations between selected polymorphisms (*CYP19*, *INSR*, *FTO*, *MC4R*) and the clinical manifestations of PCOS in a Polish female population. Methods: A total of 50 women (25 with PCOS and 25 healthy controls) were included. Genetic variants were identified using Polymerase Chain Reaction (PCR)-based methods. The frequencies of genotypes and alleles were compared between groups. Clinical symptoms such as irregular menstruation, hirsutism, acne, androgenetic alopecia, and overweight were assessed in relation to genotype. Results: No significant differences were found in genotype distributions for *CYP19*, *FTO*, *INSR*, or *MC4R* between PCOS and control groups. The *MC4R* polymorphisms showed deviations from Hardy–Weinberg equilibrium, possibly reflecting population-specific effects. Conclusions: Although most analyzed variants were not directly associated with PCOS in this cohort, the observed link between *INSR* rs1799817 and acne suggests a role in androgen-related symptoms. These findings contribute new insights to the genetic background of PCOS in Polish women and support the need for further studies combining genetic and phenotypic data in diverse populations.

## 1. Introduction

Polycystic ovary syndrome (PCOS) is one of the most common endocrine disorders in women. Its prevalence among the general female population ranges from 6 to 13%, depending on the diagnostic criteria and studied cohorts. In PCOS, numerous scientific studies have demonstrated a direct association between genetic variations and susceptibility to the disorder [1,2,3]. Individuals carrying specific genetic mutations—such as deletions, insertions, or single nucleotide polymorphisms (SNPs)—exhibit an increased risk of developing PCOS [4,5]. These genetic alterations influence various aspects of ovarian function as well as key metabolic processes, including lipid metabolism, insulin regulation, and weight control [6,7].

The occurrence of *INSR* gene polymorphisms may increase the risk of developing PCOS. This gene encodes the insulin receptor, which plays a key role in the insulin signaling pathway [8]. Disturbances in this pathway are associated with pancreatic beta cell dysfunction, leading to the development of insulin resistance [9].

Variants in the *FTO* and *MC4R* genes are associated with excess body weight, susceptibility to obesity, and increased risk of type 2 diabetes [10,11,12]. Weight gain and abdominal obesity are also characteristic features of PCOS, which exacerbate insulin resistance and hyperinsulinemia [13,14]. In addition, excess body weight increases androgen production, which consequently leads to hyperandrogenism and ovulatory dysfunction [13].

Another example of genetic influences influencing the development of PCOS are point mutations in the *CYP19* gene. They are associated with the development of estrogen-dependent disorders [15]. In PCOS, mutations or polymorphisms in *CYP19* can lead to elevated androgen levels, contributing to clinical symptoms such as hirsutism, acne, and androgenetic alopecia [16].

The aim of this study was to assess the association between selected genetic polymorphisms (*CYP19*, *MC4R*, *FTO*, and *INSR*) and the clinical manifestations of polycystic ovary syndrome in Polish women. The analysis focused on genotype distribution and allele frequencies in PCOS patients compared to healthy controls to evaluate whether these variants may contribute to the phenotypic expression of the syndrome. This study provides new data on the occurrence of these polymorphisms in the Polish population, offering insight into potential genetic susceptibility to PCOS. The present study constitutes Part II of a broader research project conducted on the same cohort of Polish women with and without PCOS. While Part I focused on dietary intake and nutritional status, the current analysis investigates genetic variants potentially associated with PCOS clinical features.

## 2. Materials and Methods

This genetic analysis was performed on the same group of 50 women (25 with PCOS, 25 healthy controls) previously described in Part I of this study, which focused on dietary intake and body composition. All participants were recruited as part of a comprehensive research project assessing both nutritional and genetic factors involved in PCOS between January 2024 and June 2024.

All participants underwent genetic analysis to assess selected polymorphisms in the *CYP19*, *MC4R*, *FTO* and *INSR* genes. As part of the study, they attended a visit to the Dietitian Service (University of Life Sciences in Lublin, Poland), where saliva samples were collected for genetic testing. During sample collection, participants were assigned to groups marked as GB (study group) for women with PCOS and GC (control group) for healthy controls.

Before participating in the study, all women were informed about the purpose of the research, the safety procedures, and the protection of their personal data. They provided written informed consent before enrollment.

The study was conducted in accordance with the Declaration of Helsinki and was approved by the University Ethics Committee for Research Involving Human Subjects (University of Life Sciences in Lublin) on 18 October 2023, approval no. UKE/10/23. All data were fully anonymized prior to access by the authors.

### 2.1. Assessment of the Frequency of Selected Genetic Polymorphisms

#### 2.1.1. Biological Material Collection and DNA Extraction

Biological material was collected using the commercial Oragene OG-500 kit (DNA Genotek, Ottawa, ON, Canada), which consists of saliva collection tubes designed for easy sample acquisition. This method allows for long-term storage of saliva samples at room temperature without compromising DNA integrity.

Genomic DNA (gDNA) was extracted from the collected samples using the Sherlock AX commercial kit (A&A Biotechnology, Gdańsk, Poland). The quality and quantity of nucleic acids were assessed using a Nanodrop 2000 UV spectrophotometer (Thermo Scientific, Waltham, MA, USA) and stored at −20 °C until analysis. Only DNA samples with purity ratios 260/280 > 1.8 and 260/230 > 1.8 were used in the study.

#### 2.1.2. Primer Selection and Genotyping

For this study, both custom-designed and previously published primers were used (Table 1). The primers for the following polymorphisms were taken from scientific literature:

- *CYP19* rs2470152 (NG_007982.1:g.40824C>T)—[17];
- *INSR* rs2059806 (NG_008852.2:g.132636G>A)—[18];
- *MC4R* rs17782313 (NC_000018.10:g.60183864T>C)—[19];
- *FTO* rs9939609 (NG_012969.1:g.87653T>A)—[20].

Additionally, in silico-designed primers were used for:

- *CYP19* rs2414096 (NG_007982.1:g.106017C>T)
- *INSR* rs1799817 (NG_008852.2:g.173715C>T)
- *MC4R* rs12970134 (NC_000018.10:g.60217517G>A)

Primer design was performed using NCBI Primer-BLAST [21], while the PREMIER Biosoft tool: Primer Premier 6.25 was employed for the detailed analysis of all primers. The sequences and parameters of each primer set are presented in Table 1.

#### 2.1.3. Polymerase Chain Reaction (PCR) and Sanger Sequencing

Using commercial NXT Taq PCR and Taq PCR Master Mix kits (EURX, Gdańsk, Poland), the Polymerase Chain Reaction (PCR) was carried out on a SimpliAmp^TM^ Thermal Cycler (Applied Biosystem, Thermo Scientific, Waltham, MA, USA) in accordance with the manufacturer’s instructions (Table S1). PCR amplification was performed under the following conditions: an initial denaturation at 95 °C for 5 min, followed by 35 cycles of denaturation at 96 °C for 5 s, primer annealing at 55–59 °C for 25 s, and extension at 68 °C for 15 s. For one minute, the last extension step was carried out at 72 °C (Table S2).

The Sanger technique was used to sequence the DNA in the following step. The resulting sequence data files were analyzed using FinchTV 1.4 (Geospiza, Denver, CO, USA. https://digitalworldbiology.com/FinchTV (on 10 June 2024)) and compared with the previously referenced sequences.

### 2.2. Characteristics of PCOS Symptoms

Data on clinical symptoms of polycystic ovary syndrome were collected from medical history and medical records provided by participants previously diagnosed according to the Rotterdam criteria. The presence of the following symptoms was analyzed: irregular menstruation, hirsutism, acne, androgenetic alopecia, and excess body weight. Each symptom was assessed qualitatively (present/absent) and then correlated with the presence of selected genetic polymorphisms (*CYP19*, *MC4R*, *FTO*, *INSR*). The aim of the analysis was to determine whether the presence of specific genetic variants is associated with a higher frequency of specific clinical symptoms of PCOS.

### 2.3. Statistical Analysis

Chi-square (χ^2^) tests were used to analyze the genotypes and their association with the occurrence of PCOS symptoms, including the test of independence and the test of concordance. The test of independence examined whether the genotype distribution depended on the group (PCOS vs. healthy), while the test of concordance compared the observed genotype distribution with the expected distribution according to the Hardy–Weinberg law.

The Hardy–Weinberg test was also used to assess whether the observed genotype frequencies within each group matched the expected frequencies, which helps detect any deviations from genetic equilibrium.

A significance level of *p* < 0.05 was adopted for the statistical analyses. Due to the large number of comparisons (approximately 30 tests), Bonferroni correction was applied to reduce the risk of Type I errors (false positives). After correction, the significance threshold was adjusted accordingly (e.g., *p* < 0.00167).

Additionally, for selected analyses, a post-hoc power analysis of the chi-square tests was performed at a significance level of α = 0.05 and degrees of freedom df = 2 using the G*Power 3.1 software.

Additionally, logistic regression was performed to assess the influence of clinical variables (irregular menstruation or ovulation, hirsutism, and BMI) on the likelihood of developing PCOS. BMI was included in the model as a control variable (covariate) to assess the independent influence of other characteristics.

## 3. Results

### 3.1. Characteristics of Groups

The basic anthropometric characteristics of the study and control groups are presented in detail in Part I of this research project [22]. Briefly, no significant differences were observed in age or height between groups. However, body weight (*p* = 0.00634), fat mass index (FMI; *p* = 0.0232), and waist circumference (*p* = 0.00328) were significantly higher in the PCOS group. Although visceral adipose tissue (VAT) levels were also higher in women with PCOS, this difference was not statistically significant (*p* = 0.07186). These variables were considered in the context of genotype–phenotype associations discussed below.

### 3.2. The Occurrence of Selected Genetic Polymorphisms in the Studied Women

The statistical analysis was based on the sequencing results, which were the basis for further calculations for *CYP19* (Table S3), *INSR* (Table S4), *MC4R* (Table S5), and *FTO* (Table S6). The Chi-square test for independence between the control group (GC) and the PCOS group (BG) was performed for the *CYP19* genotypes: rs2470152, rs241409; *INSR*: rs1799817, *rs2059806*; *MC4R*: rs12970134, rs17782313; *FTO* rs9939609; and Fisher’s exact test (Table 2).

There was no statistically significant difference in the distribution of genotypes between the control group and the PCOS group in the individual genes studied. Also, for Fisher’s exact test, the *p*-values were significantly greater than the assumed level of significance, and there was no statistically significant evidence that the genotypes were associated with the occurrence of PCOS. These results indicated that there was no difference in the frequency of genotypes between the control group and the PCOS group. The chi-square test of agreement was used in each group to check whether the observed genotypes fit the expected distribution. The expected distribution would assume that everyone has the same frequency in each group. The results for the chi-square test of agreement are presented in Table 3.

The *p*-values in the genes *CYP19*, *INSR*, *FTO* for both the control and study groups were greater than the assumed significance level of 0.05, which indicates that there is no significant deviation from the expected distribution if the genotypes were evenly distributed in each group. By contrast, for the *MC4R* rs12970134 gene, the *p*-value = 0.012 in the chi-square agreement test for the control group and *p* = 0.0003 in the study group indicated that there was a statistically significant difference between the observed and expected values for the genotypes GG, GA, AA in these groups.

Similarly for *MC4R* rs17782313 in the chi-square agreement test, the *p*-value in the control group was 0.01, indicating a statistically significant difference between the observed and expected frequencies of the genotypes TT, CT, and CC. This suggested that the observed genotype distribution was significantly different from the expected distribution. In addition, the *p*-value of 0.002 in the study group also indicated a statistically significant difference. This indicated that the observed genotype distribution was also significantly different from the expected distribution. The last test performed was to conduct a convergence analysis of the distributions, i.e., to check whether the observed genotype frequencies in both groups were consistent with the expected Hardy–Weinberg distribution (Table 4).

Based on the obtained data for the *CYP19*, *INSR*, and *FTO* genes in both the control and study groups, the observed values were close to the expected values, which suggests that there was no significant difference between them. In the control group, the distribution of genotypes was close to Hardy–Weinberg equilibrium. The deviations were small and could result from random fluctuations. In the case of *MC4R* rs12970134, the analysis of the convergence of the distributions showed differences between the observed and expected values for the GG, GA and AA genotypes in both the control and study groups, which suggested that the distribution of genotypes was not consistent with the distribution expected based on Hardy–Weinberg equilibrium (Table 4). The frequency of the G allele was higher in the study group (0.72) than in the control group (0.54), while the frequency of the A allele was clearly lower in the study group (0.28) than in the control group (0.46). The GA genotype was rare in the study group (0.08) compared to the control group (0.20). GG was more prevalent in the study group (0.68) than in the control group (0.44). AA was more common in the study group (0.24) than in the control group (0.36), but the expected values were very different.

### 3.3. Genotype–Phenotype Correlations in Women with PCOS

In this section, we analyze the relationship between the occurrence of selected clinical symptoms of polycystic ovary syndrome and the presence of specific genetic polymorphisms in the *CYP19*, *INSR*, *MC4R* and *FTO* genes. The five most frequently reported symptoms were analyzed: irregular menstruation, hirsutism, acne, androgenetic alopecia, and excessive body weight. The aim was to determine whether the frequency of individual genetic variants differs significantly depending on the presence of specific clinical symptoms of PCOS.

Due to the relatively high number of genotype–phenotype comparisons (approximately 30 tests), the Bonferroni correction was applied to adjust the significance threshold and reduce the risk of Type I errors. After correction, a stricter significance threshold of *p* < 0.00167 was used when interpreting the results.

The study assessed the relationship between the presence of selected *CYP19* gene variants (rs2414096 and rs2470152) (Table 5) and the occurrence of clinical symptoms of PCOS. For both analyzed polymorphisms, the genotypes were divided into two groups: the presence of the T allele (TT + CT genotypes) and homozygous CC. In none of the cases were statistically significant differences found in the frequency of symptoms such as irregular menstruation, hirsutism, acne, androgenetic alopecia, or excess body weight (all *p* > 0.05). In both analyses, a similar percentage distribution of genotypes was observed—the T allele occurred with a similar frequency in women with and without symptoms. Despite the observed trend of a more frequent occurrence of the T allele in symptomatic groups (e.g., 83.3% for androgenetic alopecia in rs2414096), these differences did not reach the level of statistical significance. This may indicate a lack of a clear influence of these *CYP19* polymorphisms on the phenotypic features of PCOS in the studied group or indicate the need to conduct further studies on a larger sample.

The analysis of the association between *INSR* gene variants rs1799817 (Table 6) and rs2059806 (Table 7) and the occurrence of clinical symptoms of polycystic ovary syndrome (PCOS) showed, in most cases, no significant statistical differences (*p* > 0.05).

For rs1799817, no statistically significant correlations were observed with symptoms such as menstrual irregularities, hirsutism, androgenetic alopecia, or excess body weight. However, a significant difference was observed for acne (*p* = 0.00839): women carrying the CC genotype were more likely to report this symptom (95.0%) than women carrying the T allele (5.0%). Nevertheless, after applying the Bonferroni correction for multiple testing, this association did not remain statistically significant, indicating that the result could be a false positive due to multiple comparisons.

For the rs2059806 polymorphism, there was also no statistically significant relationship between genotype and the symptoms analyzed. Although percentage differences in allele distribution were noted in several cases—including acne and excessive body weight—these did not reach the level of significance (*p* = 0.26322 and *p* = 0.20042).

In the analysis of the association between the *MC4R* rs12970134 polymorphism and the presence of clinical symptoms of PCOS, no statistically significant relationships were found (all *p* > 0.05) (Table 8).

For irregular menstruation, the presence of the A allele (AA + AG genotypes) was found in 33.3% of women with the symptom and in 53.8% of women without the symptom (*p* = 0.14433). Similar results were reported for hirsutism (35.0% vs. 50.0%), acne (50.0% vs. 40.0%), and androgenetic alopecia (50.0% vs. 43.2%). For the tendency toward excessive weight gain, carriers of the A allele accounted for 33.3% in the symptomatic group and 51.7% in the asymptomatic group.

Despite some percentage differences in the distribution of genotypes, these did not reach the level of statistical significance in any of the analyzed symptoms. These results suggest no clear association between the *MC4R* rs12970134 polymorphism and the clinical presentation of PCOS in the study population of women. Further studies on larger groups are needed to further assess the possible impact of this genetic variant.

In the analysis of the association between the *MC4R* rs17782313 polymorphism and clinical symptoms of PCOS, no statistically significant differences were found in any of the cases studied (all *p* > 0.05) (Table 9).

The presence of the C allele (CC + CT genotypes) was similarly represented among women with and without symptoms. For irregular menstruation, the frequencies were 33.3% and 38.5%, respectively (*p* = 0.70586). Similar results were also reported for hirsutism (30.0% vs. 40.0%), acne (45.0% vs. 30.0%), and androgenetic alopecia (50.0% vs. 34.1%). For the tendency towards excessive weight gain, the frequency of the C allele among symptomatic women was 28.6% and 41.4% in the asymptomatic group.

Despite the noticeable percentage differences, they did not reach the level of statistical significance. The results obtained suggest no clear association between the *MC4R* rs17782313 polymorphism and the occurrence of clinical symptoms of PCOS in the study population of women.

In the analysis of the association between the *FTO* genotype and the occurrence of clinical symptoms of polycystic ovary syndrome (PCOS), no statistically significant differences (*p* > 0.05) were found for any of the symptoms analyzed (Table 10). Among women reporting irregular menstruation, hirsutism, acne, androgenetic alopecia, and a tendency toward excessive weight gain, the variant with the presence of the A allele (AA + AT genotypes) dominated.

The highest percentage of allele A carriers was found among women with androgenetic alopecia (83.3%) and irregular menstruation and hirsutism (80% each), but these values were similar to the percentages in the asymptomatic groups and did not reach the level of statistical significance. For each symptom, the frequency of the TT genotype remained low (16.7–26.7%).

These results may indicate that in the analyzed population group, there is no clear association between the rs9939609 variant of the *FTO* gene and the phenotypic symptoms of PCOS, although noticeable percentage differences may require confirmation in studies with a larger sample.

### 3.4. Logistic Regression Analysis

A binary logistic regression analysis was performed to examine the relationship between selected clinical variables and the probability of having PCOS (Table 11). The model included three predictors: irregular menstruation or anovulation, hirsutism, and BMI. BMI was included as a covariate to adjust for its potential confounding effect.

The logistic model showed excellent overall fit, as evidenced by low deviance (12.997, df = 47), a scaled deviance < 1, and high values of pseudo R^2^ (Nagelkerke R^2^ = 0.901). ROC curve analysis yielded an AUC above 0.9, indicating very good discriminatory ability.

Two clinical features—irregular menstruation and hirsutism—were found to be very strong and statistically significant predictors of PCOS (*p* < 0.001). BMI showed a positive but non-significant association (*p* = 0.133). After applying Bonferroni correction for three predictors (adjusted significance threshold: *p* < 0.0167), irregular menstruation and hirsutism remained highly significant, while BMI did not.

These results suggest that the presence of irregular menstruation and hirsutism greatly increases the probability of PCOS, independent of BMI.

## 4. Discussion

This study aimed to evaluate the association between selected gene polymorphisms (*CYP19*, *INSR*, *FTO*, *MC4R*) and polycystic ovary syndrome in a group of Polish women. The analysis included both genotype distribution between PCOS and control groups and the relationship between specific variants and clinical symptoms such as irregular menstruation, hirsutism, acne, androgenetic alopecia, and excess body weight.

No significant differences were observed in the distribution of *CYP19*, *INSR*, and *FTO* genotypes between groups, and neither Fisher’s nor chi-square tests confirmed their association with PCOS. For *MC4R* rs12970134 and rs17782313, significant deviations from Hardy–Weinberg equilibrium were found in both groups, possibly due to sample size limitations or selection bias. Notably, the G allele of *MC4R* rs12970134 was more frequent in the PCOS group, suggesting a potential trend.

An exception was the *INSR* rs1799817 polymorphism, which initially showed a significant association with acne (*p* = 0.008), suggesting a potential involvement in hyperandrogenic pathways. However, after applying the Bonferroni correction for multiple comparisons, this association did not remain statistically significant, indicating that the finding may represent a false-positive result and requires further investigation in larger cohorts. In the studies by Ajmal et al. [23], the multifactorial nature of PCOS and the role of genetic variants related to insulin metabolism and hormonal regulation were highlighted. The *INSR* gene encodes the insulin receptor, which mediates insulin signaling and is essential for glucose homeostasis and metabolic balance. Variations in this gene can lead to altered receptor function or expression, contributing to insulin resistance—a common feature in PCOS [23]. Insulin resistance results in compensatory hyperinsulinemia, which can stimulate ovarian theca cells to produce increased amounts of androgens. Elevated androgen levels promote clinical manifestations of hyperandrogenism, including acne, hirsutism, and androgenic alopecia [24]. Although our study did not include direct measurements of insulin levels or androgen profiles, the genotype–phenotype correlation is biologically plausible, supported by extensive literature linking INSR gene variants to insulin resistance and hyperandrogenic symptoms [25,26].

A study by Hagde et al. [27] in a population of women from the Karnataka region of India found no significant association between the *CYP19* rs2414096 polymorphism and the risk of polycystic ovary syndrome. Although the GG genotype was more common in PCOS patients and GA predominated in the control group, these differences did not reach the level of statistical significance. Importantly, however, the authors observed that the GG genotype was associated with a higher LH/FSH ratio, which may reflect reduced aromatase activity and secondary hyperandrogenism. The results of our study in a population of Polish women are consistent with the above observations—also, there were no significant differences in the distribution of rs2414096 genotypes between the PCOS group and controls. This may suggest that the rs2414096 polymorphism alone is not a determinant of PCOS risk but may influence the clinical expression of some symptoms of the syndrome through hormonal mechanisms.

Zhang et al. [17], analyzing Chinese women, found no significant difference in the genotype distribution of *CYP19* rs2470152 between PCOS patients and controls, which aligns with our findings in a Polish population. However, they reported notable hormonal differences between genotypes, particularly a reduced E2/T ratio in individuals with the TC genotype. Although our study did not assess hormone levels, their results suggest that rs2470152 may influence aromatase activity and contribute to the hormonal phenotype of PCOS. These findings highlight the need for further functional studies to explore the clinical relevance of this SNP in PCOS.

The results of a large-scale genome-wide association study (GWAS) conducted by Gorsic et al. [28] highlight the significant involvement of multiple genes—such as *FTO*, *INSR*, and *MC4R*—in the pathogenesis of polycystic ovary syndrome (PCOS), regardless of the diagnostic criteria applied (Rotterdam, NIH, or AES). In our study, no statistically significant differences in genotype distribution for *FTO* and *MC4R* were observed between the PCOS and control groups, which may be attributed to the limited sample size and genetic specificity of the studied population. However, the observed association between the *INSR* rs1799817 polymorphism and the presence of clinical symptoms such as acne may suggest the involvement of this variant in metabolic disturbances, potentially leading to secondary hyperandrogenism. This finding is consistent with the results of Gorsic et al. [28], who demonstrated that disruptions in the insulin signaling pathway are a key component of PCOS pathophysiology and are closely linked to the manifestation of clinical symptoms.

The logistic regression analysis confirmed that irregular menstruation and hirsutism are the most important clinical predictors of PCOS, independently of BMI. These associations remained highly significant even after correction for multiple comparisons, reinforcing their diagnostic value. Although BMI showed a moderate positive trend, its association with PCOS was not statistically significant in our model. This suggests that clinical signs, rather than anthropometric indicators, may play a more decisive role in the diagnostic evaluation of PCOS in this population.

In our study, there were no significant differences in the frequency of *MC4R* rs12970134, rs17782313 and *FTO* rs9939609 genotypes between women with PCOS and controls. There were also no significant correlations between the genetic variants analyzed and clinical symptoms of PCOS. As shown in Part 1 of this study [21], women with PCOS were characterized by significantly higher body weight, FMI and waist circumference, confirming the presence of an unfavorable metabolic phenotype. Despite the lack of direct genotype–phenotype correlations in our analysis, previous studies [19] have shown an association of *FTO* rs9939609 with PCOS risk, particularly in interaction with *MC4R* rs17782313, although this relationship was modified by BMI. Our results, although different, are nevertheless a valuable contribution to the description of the population of Polish women with PCOS. The comprehensive analysis of genotypes in combination with body composition analysis and the assessment of clinical symptoms enriches the knowledge of potential determinants of the syndrome and points to directions for further research taking into account genetic–environmental interactions.

### Study Limitations

Although the sample size (n = 25 in each group) may be considered a limitation in the context of complex genetic studies, our post-hoc analyses showed that the power of the test for key comparisons—particularly those related to MC4R polymorphisms—was sufficient (>0.8) to detect medium to large effects. However, further studies with larger cohorts are necessary to confirm these results.

A major limitation of our study is the lack of hormonal and metabolic biomarker data, which could provide functional insights into the effects of the polymorphisms studied. This absence restricts the interpretation of the genetic associations, as we cannot correlate genotype data with endocrine or metabolic profiles that are crucial in PCOS pathophysiology. Consequently, the biological mechanisms behind the observed genetic variants remain speculative. Furthermore, the relatively small sample size may limit the generalizability of the findings despite acceptable statistical power for detecting medium to large effects. To overcome these limitations, future research should incorporate multi-omics approaches—combining genetic, epigenetic, transcriptomic, and proteomic data—with comprehensive clinical and environmental profiling. Such integrative studies would offer a deeper understanding of the complex molecular pathways involved in PCOS and improve the identification of clinically relevant biomarkers.

## 5. Conclusions

Our study provides important genetic data on the association between selected gene polymorphisms and PCOS specifically in Polish women, a population that is underrepresented in current genetic research on PCOS. Although polymorphisms in genes such as *CYP19*, *INSR*, *FTO*, and *MC4R* have been widely studied in larger European cohorts and meta-analyses, our investigation adds value by focusing on a relatively genetically homogeneous Polish cohort, which may help refine population-specific risk profiles.

Our findings revealed no significant differences in the distribution of the analyzed gene polymorphisms between PCOS patients and controls, consistent with previous studies in other European populations. This genetic similarity suggests that the impact of these polymorphisms on PCOS susceptibility may be broadly conserved across European populations, including the Polish population. Therefore, while our results do not identify novel genetic risk factors unique to Polish women, they contribute to the growing evidence of genetic consistency in PCOS susceptibility loci across Europe and emphasize the importance of including diverse but related populations in genetic research.

Given the multifactorial nature of PCOS, future research should focus on larger, ethnically diverse cohorts to validate these associations and unravel population-specific genetic effects. Moreover, integrating environmental factors—including dietary habits, physical activity, and exposure to endocrine disruptors—will be crucial to better understand gene–environment interactions that modulate PCOS risk and phenotype expression.

The inclusion of comprehensive biochemical profiling, such as hormone levels (androgens, insulin and LH/FSH ratio), metabolic markers, and inflammatory mediators, would enable a more detailed exploration of how genetic polymorphisms influence metabolic and endocrine dysfunctions in PCOS. Longitudinal studies could further clarify how these interactions evolve over time and affect disease progression and response to treatment.

This study’s multidimensional design—combining genetic polymorphism analysis, clinical symptom assessment, body composition evaluation through bioelectrical impedance analysis (BIA) and detailed dietary intake profiling (reported in the first part of the study)—provides a robust framework for personalized medicine approaches. Such comprehensive assessments can help identify distinct PCOS phenotypes and inform tailored interventions, potentially improving clinical outcomes and quality of life.

Ultimately, advancing our understanding of the complex genetic and environmental underpinnings of PCOS will facilitate the development of more effective prevention, diagnostic, and therapeutic strategies, moving beyond one-size-fits-all approaches to precision health care for women affected by this syndrome.

## Figures and Tables

**Table 1:** Parameters of primers and length of PCR products (own compilation based on https://www.premierbiosoft.com/primerdesign/index.html) (accessed on 10 June 2024 = 1, https://www.ncbi.nlm.nih.gov/tools/primer-blast/) (accessed on 10 June 2024).

SNP	Starter Sequence (5′→3′)	Starter Length (bp)	Product Length (bp)	Tm (°C)	GC (%)	Cross Dimer	Self Dimer	Hairpin
*CYP19 *^a^ rs2470152	CTGCCTTTGAGGAGCTTACTGT CTTCTCTGGCTTTCCCCTCT	22 20	278	57.73 55.61	50 55	−2.9	−3.0 0.0	−1.3 0.0
*CYP19* rs2414096	TTGTTCCCTGGACGCATGTT CTTTTGGTTTGAGTGCCCCG	20 20	350	57.1 56.97	50 55	−2.0	−2.3 0.0	0.0 0.0
*INSR* rs1799817	GGTGAAGACGGTCAACGAGT AGCTTAGTGGAGTGAGGGGT	20 20	222	59.97 59.88	55.00 55.00	−0.9	−1.1 −3.0	−0.9 0.0
*INSR *^b^ rs2059806	CGGTCTTGTAAGGGTAACTG GAATTCACATTCCCAAGACA	20 20	322	55.51 53.47	50.00 40.00	−5.3	−0.5 −4.3	0.0 −1.5
*MC4R* rs12970134	GTCAGGTCATTGGAAGAGGCT ATGCTTGCCCTGCTAGGTTG	21 20	385	56.9 57.61	52.38 55.00	−3.0	0.0 −1.6	0.0 −1.3
*MC4R *^c^ rs17782313	AGGAAACAGCAGGGATAGGG TGCTGAGACAGGTTCATAAAAG	20 23	407	55.66 54.74	55.00 39.13	−4.9	0.0 −1.1	0.0 −1.1
*FTO *^d^ rs9939609	AGGAGAGGAGAAAGTGAGCT TGTTCAAGTCACACTCAGCCTC	20 22	504	57.38 60.48	50.00 50.00	−2.9	−3.0 −1.0	0.0 −1.0

^a^ [17], ^b^ [18], ^c^ [19], ^d^ [20].

**Table 2:** Results of the chi-square test and Fisher’s exact test for genes *CYP19*, *INSR*, *MC4R*, and *FTO* for GB—the study group—and GC—the control group.

Gene Polymorphism	Genotype	Observed Value	Expected Value	χ^2^	*p* ^1^	*p* ^2^
*CYP19* rs2470152	CC	GC: 8	GC: 7.35	1.814	0.404	0.414
		GB: 7	GB: 7.65			
	TT	GC: 3	GC: 4.90			
		GB: 7	GB: 5.10			
	CT	GC: 13	GC: 11.76			
		GB: 11	GB: 12.24			
*CYP19* rs2414096	CC	GC: 5	GC: 6	0.556	0.757	0.726
		GB: 7	GB: 6			
	CT	GC: 10	GC: 10			
		GB: 10	GB: 10			
	TT	GC: 10	GC: 9			
		GB: 8	GB: 9			
*INSR* rs1799817	CC	GC: 18	GC: 18	1.077	0.584	1.0
		GB: 18	GB: 18			
	CT	GC: 7	GC: 6.5			
		GB: 6	GB: 6.5			
	TT	GC: 0	GC: 0.5			
		GB: 1	GB: 0.5			
*INSR* rs2059806	GG	GC: 18	GC: 18.5	0.118	0.943	1.0
		GB: 19	GB: 18.5			
	AA	GC: 1	GC: 1.0			
		GB: 1	GB: 1.0			
	GA	GC: 6	GC: 5.5			
		GB: 5	GB: 5.5			
*MC4R* rs12970134	GG	GC: 11	GC: 14	3.171	0.205	0.207
		GB: 17	GB: 14			
	GA	GC: 5	GC: 3.5			
		GB: 2	GB: 3.5			
	AA	GC: 9	GC: 7.5			
		GB: 6	GB: 7.5			
*MC4R* rs17782313	TT	GC: 15	GC: 16	2.125	0.346	0.485
		GB: 17	GB: 16			
	CC	GC: 2	GC: 1.0			
		GB: 0	GB: 1.0			
	TC	GC: 8	GC: 8.0			
		GB: 8	GB: 8.0			
*FTO* rs9939609	TT	GC: 7	GC: 6.5	0.11	0.945	1.0
		GB: 6	GB: 6.5			
	AA	GC: 5	GC: 5.0			
		GB: 5	GB: 5.0			
	TA	GC: 13	GC: 13.5			
		GB: 14	GB: 13.5			

*p*^1^—*p*-value from the chi-square test; *p*^2^—*p*-value from Fisher’s exact test.

**Table 3:** Results of the chi-square test of agreement for genes *CYP19*, *INSR*, *MC4R*, *FTO*.

***CYP19*** **rs2470152**				
**Control Group**				
**Genotype**	**Observed value**	**Expected value**	**χ^2^**	***p***
CC	8	8.76	0.421	0.810
TT	3	3.76		
CT	13	11.48		
**Study Group**				
**Genotype**	**Observed value**	**Expected value**	**χ^2^**	***p***
CC	7	6.25	0.360	0.835
TT	7	6.25		
CT	11	12.25		
***CYP19*** **rs2414096**				
**Control Group**				
**Genotype**	**Observed value**	**Expected value**	**χ^2^**	***p***
CC	5	4.0	0.694	0.707
CT	10	12.0		
TT	10	9.0		
**Study Group**				
**Genotype**	**Observed value**	**Expected value**	**χ^2^**	***p***
CC	7	5.76	0.987	0.610
CT	10	12.48		
TT	8	6.76		
***INSR*** **rs1799817**				
**Control Group**				
**Genotype**	**Observed value**	**Expected value**	**χ^2^**	***p***
CC	18	18.49	0.663	0.718
CT	7	6.02		
TT	0	0.49		
**Study Group**				
**Genotype**	**Observed value**	**Expected value**	**χ^2^**	***p***
CC	18	17.64	0.287	0.866
CT	6	6.72		
TT	1	0.64		
***INSR*** **rs2059806**				
**Control Group**				
**Genotype**	**Observed value**	**Expected value**	**χ^2^**	***p***
**GG**	18	17.64	0.287	0.866
AA	1	0.64		
GA	6	6.72		
**Study Group**				
**Genotype**	**Observed value**	**Expected value**	**χ^2^**	***p***
GG	19	18.49	0.718	0.698
AA	1	0.49		
GA	5	6.02		
***MC4R*** **rs12970134**				
**Control Group**				
**Genotype**	**Observed value**	**Expected value**	**χ^2^**	***p***
GG	11	7.29	8.923	0.012
GA	5	12.42		
AA	9	5.29		
**Study Group**				
**Genotype**	**Observed value**	**Expected value**	**χ^2^**	***p***
GG	17	12.96	16.064	0.0003
GA	2	10.08		
AA	6	1.96		
***MC4R*** **rs17782313**				
**Control Group**				
**Genotype**	**Observed value**	**Expected value**	**χ^2^**	***p***
TT	15	14.44	9.13	0.01
CC	2	9.12		
TC	8	1.44		
**Study Group**				
**Genotype**	**Observed value**	**Expected value**	**χ^2^**	***p***
TT	17	17.64	13.00	0.002
CC	0	6.72		
TC	8	0.64		
***FTO*** **rs9939609**				
**Control Group**				
**Genotype**	**Observed value**	**Expected value**	**χ^2^**	***p***
TT	7	7.29	0.03	0.98
AA	5	5.29		
TA	13	12.42		
**Study Group**				
**Genotype**	**Observed value**	**Expected value**	**χ^2^**	***p***
TT	6	6.76	0.19	0.911
AA	5	5.76		
TA	14	12.48		

**Table 4:** Results of the Hardy–Weinberg convergence analysis for the genes *CYP19*, *INSR*, *MC4R*, *FTO*.

***CYP19*** **rs2470152**			
**Control Group**			
**Allele/Genotype**	**Frequency**	**Observed value**	**Expected value**
C	0.6		
T	0.4		
CC	0.33	8	8.76
TT	0.13	3	3.76
CT	0.54	13	11.48
**Study Group**			
**Allele/Genotype**	**Frequency**	**Observed value**	**Expected value**
C	0.5		
T	0.5		
CC	0.28	7	6.25
TT	0.28	7	6.25
CT	0.44	11	12.5
***CYP19*** **rs2414096**			
**Control Group**			
**Allele/Genotype**	**Frequency**	**Observed value**	**Expected value**
C	0.4		
T	0.6		
CC	0.20	5	4
CT	0.40	10	12
TT	0.40	10	9
**Study Group**			
**Allele/Genotype**	**Frequency**	**Observed value**	**Expected value**
C	0.48		
T	0.52		
CC	0.28	7	5.76
CT	0.40	10	12.48
TT	0.32	8	6.76
***INSR*** **rs1799817**			
**Control Group**			
**Allele/Genotype**	**Frequency**	**Observed value**	**Expected value**
C	0.86		
T	0.14		
CC	0.72	18	18.49
CT	0.28	7	6.02
TT	0.00	0	0.49
**Study Group**			
**Allele/Genotype**	**Frequency**	**Observed value**	**Expected value**
C	0.84		
T	0.16		
CC	0.72	18	17.64
CT	0.24	6	6.72
TT	0.04	1	0.64
***INSR*** **rs2059806**			
**Control Group**			
**Allele/Genotype**	**Frequency**	**Observed value**	**Expected value**
G	0.84		
A	0.16		
GG	0.72	18	17.64
AA	0.04	1	0.64
GA	0.24	6	6.72
**Study Group**			
**Allele/Genotype**	**Frequency**	**Observed value**	**Expected value**
G	0.86		
A	0.14		
GG	0.76	19	18.49
AA	0.04	1	0.49
GA	0.20	5	6.02
***MC4R*** **rs12970134**			
**Control Group**			
**Allele/Genotype**	**Frequency**	**Observed value**	**Expected value**
G	0.54		
A	0.46		
GG	0.44	11	7.29
GA	0.20	5	12.42
AA	0.36	9	5.29
**Study Group**			
**Allele/Genotype**	**Frequency**	**Observed value**	**Expected value**
G	0.72		
A	0.28		
GG	0.68	17	12.96
GA	0.08	2	10.08
AA	0.24	6	1.96
***MC4R*** **rs17782313**			
**Control Group**			
**Allele/Genotype**	**Frequency**	**Observed value**	**Expected value**
T	0.76		
C	0.24		
TT	0.58	15	14.44
CC	0.06	2	1.44
TC	0.36	8	9.12
**Study Group**			
**Allele/Genotype**	**Frequency**	**Observed value**	**Expected value**
T	0.84		
C	0.16		
TT	0.70	17	17.64
CC	0.03	0	0.64
TC	0.27	8	6.72
***FTO*** **rs9939609**			
**Control Group**			
**Allele/Genotype**	**Frequency**	**Observed value**	**Expected value**
**T**	0.54		
A	0.46		
**TT**	0.29	7	7.29
**AA**	0.21	5	5.29
**TA**	0.50	13	12.42
**Study Group**			
**Allele/Genotype**	**Frequency**	**Observed value**	**Expected value**
T	0.52		
A	0.48		
TT	0.27	6	6.76
AA	0.23	5	5.76
TA	0.50	14	12.48

**Table 5:** The relationship between PCOS symptoms and *CYP19* rs2414096 * and rs2470152 * polymorphism in the analyzed group of women.

Clinical Symptom	CYP19 Genotype	Symptom Presence—YES (n, %)	Symptom Presence—NO (n, %)	*p* (Chi^2^)
**Irregular menstruation**	T (TT + CT)	16 (66.7%)	19 (73.1%)	0.62119
	CC	8 (33.3%)	7 (26.9%)	
**Hirsutism**	T (TT + CT)	15 (75.0%)	20 (66.7%)	0.52873
	CC	5 (25.0%)	10 (33.3%)	
**Acne**	T (TT + CT)	13 (65.0%)	22 (73.3%)	0.52873
	CC	7 (35.0%)	8 (26,7%)	
**Androgenetic alopecia**	T (TT + CT)	5 (83.3%)	30 (68.2%)	0.77572
	CC	1 (16.7%)	14 (31.8%)	
**Excessive body weight**	T (TT + CT)	16 (76.2%)	19 (65.5%)	0.41630
	CC	5 (23.8%)	10 (34.5%)	

* As the distribution of outcomes and corresponding *p*-values were identical for rs2414096 and rs2470152, the results have been combined and presented together.

**Table 6:** The relationship between PCOS symptoms and *INSR* rs1799817 polymorphism in the analyzed group of women.

Clinical Symptom	*INSR* Genotype	Symptom Presence—YES (n, %)	Symptom Presence—NO (n, %)	*p* (Chi^2^)
**Irregular menstruation**	T (TT + CT)	6 (25.0%)	8 (30.8%)	0.64989
	CC	18 (75.0%)	18 (69.2%)	
**Hirsutism**	T (TT + CT)	6 (30.0%)	8 (26.7%)	0.79705
	CC	14 (70.0%)	22 (73.3%)	
**Acne**	T (TT + CT)	1 (5.0%)	13 (43.3%)	0.00839 *
	CC	19 (95.0%)	17 (56.7%)	
**Androgenetic alopecia**	T (TT + CT)	12 (27.3%)	2 (33.3%)	0.86150
	CC	32 (72.7%)	4 (66.7%)	
**Excessive body weight**	T (TT + CT)	6 (28.6%)	8 (27.6%)	0.93896
	CC	15 (71.4%)	21 (72.4%)	

* Statistically significant result (*p* < 0.05). The table presents *p*-values without correction for multiple testing. After applying the Bonferroni correction (significance threshold *p* < 0.00167), none of the observed differences were statistically significant.

**Table 7:** The relationship between PCOS symptoms and *INSR* rs2059806 polymorphism in the analyzed group of women.

Clinical Symptom	*INSR* Genotype	Symptom Presence—YES (n, %)	Symptom Presence—NO (n, %)	*p* (Chi^2^)
**Irregular menstruation**	A (AA + AG)	6 (25.0%)	7 (26.9%)	0.87691
	GG	18 (75.0%)	19 (73.1%)	
**Hirsutism**	A (AA + AG)	5 (25.0%)	8 (26.7%)	0.89528
	GG	15 (75.0%)	22 (73.3%)	
**Acne**	A (AA + AG)	3 (15.0%)	10 (33.3%)	0.26322
	GG	17 (85.0%)	20 (66.7%)	
**Androgenetic alopecia**	A (AA + AG)	1 (16.7%)	12 (27.3%)	0.57848
	GG	5 (83.3%)	32 (72.7%)	
**Excessive body weight**	A (AA + AG)	3 (14.3%)	10 (34.5%)	0.20042
	GG	18 (85.7%)	19 (65.5%)	

**Table 8:** The relationship between PCOS symptoms and the *MC4R* rs12970134 polymorphism in the analyzed group of women.

Clinical Symptom	*MC4R* Genotype	Symptom Presence—YES (n, %)	Symptom Presence—NO (n, %)	*p* (Chi^2^)
**Irregular menstruation**	A (AA + AG)	8 (33.3%)	14 (53.8%)	0.14433
	GG	16 (66.7%)	12 (46.2%)	
**Hirsutism**	A (AA + AG)	7 (35.0%)	15 (50.0%)	0.29519
	GG	13 (65.0%)	15 (50.0%)	
**Acne**	A (AA + AG)	10 (50.0%)	12 (40.0%)	0.48526
	GG	10 (50.0%)	18 (60.0%)	
**Androgenetic alopecia**	A (AA + AG)	3 (50.0%)	19 (43.2%)	0.90231
	GG	3 (50.0%)	25 (56.8%)	
**Excessive body weight**	A (AA + AG)	7 (33.3%)	15 (51.7%)	0.19601
	GG	14 (66.7%)	14 (48.3%)	

**Table 9:** The relationship between PCOS symptoms and the *MC4R* rs17782313 polymorphism in the analyzed group of women.

Clinical Symptom	*MC4R* Genotype	Symptom Presence—YES (n, %)	Symptom Presence—NO (n, %)	*p* (Chi^2^)
**Irregular menstruation**	C (CC + CT)	8 (33.3%)	10 (38.5%)	0.70586
	TT	16 (66.7%)	16 (61.5%)	
**Hirsutism**	C (CC + CT)	6 (30.0%)	12 (40.0%)	0.47049
	TT	14 (70.0%)	18 (60.0%)	
**Acne**	C (CC + CT)	9 (45.0%)	9 (30.0%)	0.27902
	TT	11 (55.0%)	21 (70.0%)	
**Androgenetic alopecia**	C (CC + CT)	3 (50.0%)	15 (34.1%)	0.75788
	TT	3 (50.0%)	29 (65.9%)	
**Excessive body weight**	C (CC + CT)	6 (28.6%)	12 (41.4%)	0.35173
	TT	15 (71.4%)	17 (58.6%)	

**Table 10:** The relationship between PCOS symptoms and the *FTO* rs9939609 polymorphism in the analyzed group of women.

Clinical Symptom	*FTO* Genotype	Symptom Presence—YES (n, %)	Symptom Presence—NO (n, %)	*p* (Chi^2^)
**Irregular menstruation**	A (AA + AT)	18 (75%)	20 (76.9%)	0.8736
	TT	6 (25%)	6 (23.1%)	
**Hirsutism**	A (AA + AT)	16 (80%)	22 (73.3%)	0.5887
	TT	4 (20%)	8 (26.7%)	
**Acne**	A (AA + AT)	16 (80%)	22 (73.3%)	0.58869
	TT	4 (20%)	8 (26,7%)	
**Androgenetic alopecia**	A (AA + AT)	5 (83.3%)	33 (75%)	0.65390
	TT	1 (16.7%)	11 (25%)	
**Excessive body weight**	A (AA + AT)	7 (24.1%)	22 (75.9%)	0.97859
	TT	5 (23.8%)	16 (76.2%)	

**Table 11:** Logistic regression results for clinical predictors of PCOS.

Variable	Coefficient (B)	*p*-Value	Odds Ratio (OR)	Interpretation
**Intercept**	−27.661	0.000	–	Statistically significant; not interpreted clinically
**Irregular menstruation/anovulation**	22.491	0.000	736.904	Strong, highly significant positive predictor
**BMI**	0.260	0.133	1.296	30% increase in odds per 1 unit of BMI; not statistically significant
**Hirsutism**	21.199	0.000	520.546	Strong, highly significant positive predictor

Bonferroni-adjusted significance threshold for multiple testing: *p* < 0.0167.

## Data Availability

Data is contained within the article.

## Supplementary Materials

The following supporting information can be downloaded at https://www.mdpi.com/article/10.3390/genes16070840/s1. Table S1: Composition and volume of reaction mixtures used for PCR reactions; Table S2: PCR reaction program, Table S3: Sanger sequencing results of the studied samples: GB study group (PCOS) and GC control group (non-PCOS) for *CYP19* rs2470152 and *CYP19* rs2414096; Table S4: Sanger sequencing results of the studied samples: GB study group (PCOS) and GC control group (non-PCOS) for *INSR* rs1799817 and *INSR* rs2059806, Table S5: Sanger sequencing results of the studied samples: GB study group (PCOS) and GC control group (non-PCOS) for *MC4R* rs12970134 and *MC4R* rs17782313, Table S6: Sanger sequencing results of the studied samples: GB study group (PCOS) and GC control group (non-PCOS) for *FTO* rs9939609.

## Abbreviations

The following abbreviations are used in this manuscript:

PCOS	Polycystic Ovary Syndrome
SNP	Single Nucleotide Polymorphism
*INSR*	Insulin Receptor
*FTO*	Fat Mass and Obesity-associated gene
*MC4R*	Melanocortin 4 Receptor
*CYP19*	Cytochrome P450 Family 19 Subfamily A Member 1
gDNA	Genomic DNA
PCR	Polymerase Chain Reaction
OG-500	Oragene DNA Self-Collection Kit
VAT	Visceral Adipose Tissue
FMI	Fat Mass Index
GC	Control Group
GB	Study Group
Tm	Melting Temperature
NCBI	National Center for Biotechnology Information
