# Supplementary material for: Dietary and Genetic Aspects of Polycystic Ovary Syndrome (PCOS) in Polish Women—Part II: Association of CYP19, FTO, MC4R and INSR Gene Polymorphisms with Clinical Symptoms of PCOS
Source: Genes (Basel). 2025 Jul 18;16(7):840. doi: 10.3390/genes16070840 (PMC12294189; doi:10.3390/genes16070840)
Supplement: Supplementary file 1 [file genes-16-00840-s001.zip › genes-3736862-supplementary.pdf]

Table S1. Composition and volume of reaction mixtures used for PCR

NXT Taq PCR Kit		Taq PCR Master Mix Kit (2x)	
**Ingredients**	Volume (μl)	**Ingredients**	Volume (μl)
**NXT *Taq* PCR Master Mix (2x)**	10	***Taq* PCR Master Mix (2x)**	14
**Starter *forward* (10 μM)**	1	**Starter *forward* (10 μM)**	1
**Starter *reverse* (10 μM)**	1	**Starter *reverse* (10 μM)**	1
**Nuclease-free water**	4	**Nuclease-free water**	7
**DNA**	2	**DNA**	2
**In total**	18	**In total**	25

Table S2. PCR program

**Stage**	**Temperature**	**Time**	**Number of cycles**
**Pre-denaturation**	95°C	5 minutes	1
**Denaturation**	96°C	5 seconds	35
Annealing	55°C[a] or 57°C[b] or 59°C[c]	25 seconds	
	68°C		
**Extension**		15 seconds	
**Final extension**	72°C	1 minutes	1
**Cooling**	4°C	∞	1

[a]55°C for primers *INSR* rs2059806, *MC4R* rs17782313; [b] 57° for primers *CYP19* rs2470152, *CYP19* rs2414096, *MC4R* rs12970134, [c]59°C for primers *INSR* rs1799817 and *FTO* rs9939609

Table S3. Sanger sequencing results of the studied samples: GB study group (PCOS) and GC control group (non-PCOS) for *CYP19* rs2470152 and *CYP19* rs2414096

**Patients**		**Gene**			
		***CYP19* rs2470152**		***CYP19* rs2414096**	
**Group**	**Number**	**Research code**	**Results**	**Research code**	**Results**
**Study group GB (PCOS)**	1	4.1	CT	5.1	TT
	2	4.2	CC	5.2	TT
	3	4.3	CT	5.3	CT
	5	4.5	CC	5.5	TT
	6	4.6	CC	5.6	TT
	7	4.7	TT	5.7	CC
	8	4.8	CT	5.8	CT
	9	4.9	TT	5.9	CC
	10	4.10	TT	5.10	CC
	11	4.11	TT	5.11	CC
	12	4.12	CT	5.12	CT
	13	4.13	TT	5.13	CT
	14	4.14	CC	5.14	TT
	15	4.15	CT	5.15	CT
	16	4.16	TT	5.16	CC
	17	4.17	CT	5.17	TT
	18	4.18	CC	5.18	TT
	19	4.19	CT	5.19	CT
	20	4.20	CC	5.20	TT
	21	4.21	CT	5.21	CT
	22	4.22	CT	5.22	CC
	23	4.23	TT	5.23	CC
	24	4.24	CC	5.24	CT
	25	4.25	CT	5.25	CT
	26	4.26	CT	5.26	CT
**Control group GC (non-PCOS)**	1	4.27	CT	5.27	CC
	2	4.28	TT	5.28	CC
	3	4.29	CC	5.29	CT
	4	4.30	CT	5.30	CC
	5	4.31	CT	5.31	TT
	6	4.32	TT	5.32	CT
	7	4.33	CT	5.33	TT
	8	4.34	CT	5.34	CT
	9	4.35	CT	5.35	CT
	10	4.36	CT	5.36	CT
	11	4.37	CC	5.37	TT
	12	4.38	CC	5.38	TT
	13	4.39	CT	5.39	TT
	14	4.40	CT	5.40	CC
	15	4.41	CC	5.41	TT
	16	4.42	CT	5.42	CT
	17	4.43	TT	5.43	CT

	18	4.44	CC	5.44	TT
	19	4.45	-	5.45	TT
	20	4.46	CC	5.46	TT
	21	4.47	CT	5.47	CT
	22	4.48	CC	5.48	CT
	25	4.49	CC	5.49	TT
	26	4.50	CT	5.50	CT
	27	4.51	CT	5.51	CC

Table S4. Sanger sequencing results of the studied samples: GB study group (PCOS) and GC control group (non-PCOS) for *INSR* rs1799817 and *INSR* rs2059806

**Patients**		**Gene**			
		INSR **rs1799817**		***INSR*** **rs2059806**	
**Group**	**Number**	**Research code**	**Results**	**Research code**	**Results**
**Study group GB (PCOS)**	1	1.1	CC	2.1	GG
	2	1.2	CC	2.2	AA
	3	1.3	CC	2.3	GG
	5	1.5	CC	2.5	GG
	6	1.6	CC	2.6	GG
	7	1.7	CC	2.7	GA
	8	1.8	CT	2.8	GG
	9	1.9	TT	2.9	GG
	10	1.10	CC	2.10	GA
	11	1.11	CT	2.11	GG
	12	1.12	CT	2.12	GG
	13	1.13	CT	2.13	GA
	14	1.14	CT	2.14	GA
	15	1.15	CC	2.15	GG
	16	1.16	CC	2.16	GG
	17	1.17	CC	2.17	GG
	18	1.18	CC	2.18	GG
	19	1.19	CC	2.19	GG
	20	1.20	CC	2.20	GG
	21	1.21	CC	2.21	GG
	22	1.22	CC	2.22	GG
	23	1.23	CT	2.23	GA
	24	1.24	CC	2.24	GG
	25	1.25	CC	2.25	GG
	26	1.26	CC	2.26	GG
**Control group GC (non-PCOS)**	1	1.27	CT	2.27	GA
	2	1.28	CC	2.28	GG
	3	1.29	CC	2.29	GG
	4	1.30	CC	2.30	GA
	5	1.31	CC	2.31	GG
	6	1.32	CC	2.32	GG

	7	1.33	CC	2.33	GG
	8	1.34	CC	2.34	GG
	9	1.35	CC	2.35	GG
	10	1.36	CC	2.36	GG
	11	1.37	CC	2.37	GG
	12	1.38	CT	2.38	GG
	13	1.39	CC	2.39	GG
	14	1.40	CT	2.40	GA
	15	1.41	CT	2.41	GA
	16	1.42	CC	2.42	AA
	17	1.43	CC	2.43	GG
	18	1.44	CC	2.44	GA
	19	1.45	CT	2.45	GG
	20	1.46	CT	2.46	GG
	21	1.47	CC	2.47	GG
	22	1.48	CC	2.48	GG
	25	1.49	CC	2.49	GG
	26	1.50	CC	2.50	GG
	27	1.51	CT	2.51	GA

Table S5. Sanger sequencing results of the studied samples: GB study group (PCOS) and GC control group (non-PCOS) for *MC4R* rs12970134 and *MC4R* rs17782313

**Patients**		**Gene**			
		***MC4R* rs12970134**		***MC4R* rs17782313**	
**Group**	**Number**	**Research code**	**Results**	**Research code**	**Results**
**Study group GB (PCOS)**	1	6.1	GG	7.1	TT
	2	6.2	GG	7.2	TC
	3	6.3	GG	7.3	TT
	5	6.5	AA	7.5	TC
	6	6.6	GG	7.6	TT
	7	6.7	GG	7.7	TT
	8	6.8	GA	7.8	TC
	9	6.9	AA	7.9	TT
	10	6.10	AA	7.10	TC
	11	6.11	GG	7.11	TT
	12	6.12	AA	7.12	TC
	13	6.13	GG	7.13	TT
	14	6.14	GG	7.14	TT
	15	6.15	AA	7.15	TC
	16	6.16	GG	7.16	TT
	17	6.17	AA	7.17	TC
	18	6.18	GG	7.18	TT
	19	6.19	GA	7.19	TC
	20	6.20	GG	7.20	TT
	21	6.21	GG	7.21	TT
	22	6.22	GG	7.22	TT

	23	6.23	GG	7.23	TT
	24	6.24	GG	7.24	TT
	25	6.25	GG	7.25	TT
	26	6.26	GG	7.26	TT
**Control group GC (non-PCOS)**	1	6.27	GA	7.27	TC
	2	6.28	AA	7.28	TT
	3	6.29	AA	7.29	TT
	4	6.30	AA	7.30	TT
	5	6.31	GG	7.31	TC
	6	6.32	GG	7.32	TT
	7	6.33	AA	7.33	CC
	8	6.34	GA	7.34	CC
	9	6.35	GG	7.35	TT
	10	6.36	GG	7.36	TT
	11	6.37	GA	7.37	TT
	12	6.38	GG	7.38	TT
	13	6.39	AA	7.39	TC
	14	6.40	GG	7.40	TT
	15	6.41	GG	7.41	TT
	16	6.42	GG	7.42	TT
	17	6.43	GG	7.43	TT
	18	6.44	GA	7.44	TC
	19	6.45	AA	7.45	TT
	20	6.46	AA	7.46	TC
	21	6.47	GG	7.47	TT
	22	6.48	AA	7.48	TC
	25	6.49	AA	7.49	TC
	26	6.50	GA	7.50	TC
	27	6.51	GG	7.51	TT

Table S6. Sanger sequencing results of the studied samples: GB study group (PCOS) and GC control group (non-PCOS) for *FTO* rs9939609

**Patients**		**Gene**	
		***FTO* rs9939609**	
**Group**	**Number**	**Research code**	**Results**
**Study group GB (PCOS)**	1	3.1	AA
	2	3.2	TA
	3	3.3	AA
	5	3.5	TT
	6	3.6	TA
	7	3.7	AA
	8	3.8	AA
	9	3.9	TT
	10	3.10	TA
	11	3.11	TA

	12	3.12	TT
	13	3.13	TA
	14	3.14	TT
	15	3.15	TA
	16	3.16	TA
	17	3.17	AA
	18	3.18	TA
	19	3.19	TT
	20	3.20	TA
	21	3.21	TA
	22	3.22	TA
	23	3.23	TT
	24	3.24	TA
	25	3.25	TA
	26	3.26	TA
**Control group GC (non-PCOS)**	1	3.27	TT
	2	3.28	TA
	3	3.29	TT
	4	3.30	TA
	5	3.31	TA
	6	3.32	TT
	7	3.33	TA
	8	3.34	TA
	9	3.35	TA
	10	3.36	AA
	11	3.37	TA
	12	3.38	TT
	13	3.39	AA
	14	3.40	AA
	15	3.41	TT
	16	3.42	AA
	17	3.43	TA
	18	3.44	AA
	19	3.45	TA
	20	3.46	TA
	21	3.47	TT
	22	3.48	TA
	25	3.49	TA
	26	3.50	TA
	27	3.51	TT
